# Genome-wide characterization of *SDR* gene family and its potential role in seed dormancy of *Brassica napus* L.

**DOI:** 10.1186/s12870-023-04700-2

**Published:** 2024-01-02

**Authors:** Fugui Zhang, Tianhua Chen, Nian Liu, Xinzhe Hou, Ling Wang, Qingao Cai, Rui Li, Xingzhi Qian, Hong Xu, Zonghe Zhu, Wenyin Zheng, Yan Yu, Kejin Zhou

**Affiliations:** College of Agronomy, Anhui Agricultural University, 130, Changjiang West Road, Hefei, Anhui 230036 China

**Keywords:** Rapeseed **(***Brassica napus* L.), *SDR* gene family, Seed dormancy, Pre-harvest sprouting, Gene expression

## Abstract

Rapeseed (*Brassica napus* L.) with short or no dormancy period are easy to germinate before harvest (pre-harvest sprouting, PHS). PHS has seriously decreased seed weight and oil content in *B. napus.* Short-chain dehydrogenase/ reductase (*SDR*) genes have been found to related to seed dormancy by promoting ABA biosynthesis in rice and *Arabidopsis*. In order to clarify whether *SDR* genes are the key factor of seed dormancy in *B. napus*, homology sequence blast, protein physicochemical properties, conserved motif, gene structure, cis-acting element, gene expression and variation analysis were conducted in present study. Results shown that 142 *BnaSDR* genes, unevenly distributed on 19 chromosomes, have been identified in *B. napus* genome. Among them, four *BnaSDR* gene clusters present in chromosome A04、A05、C03、C04 were also identified. These 142 *BnaSDR* genes were divided into four subfamilies on phylogenetic tree. Members of the same subgroup have similar protein characters, conserved motifs, gene structure, cis-acting elements and tissue expression profiles. Specially, the expression levels of genes in subgroup A, B and C were gradually decreased, but increased in subgroup D with the development of seeds. Among seven higher expressed genes in group D, six *BnaSDR* genes were significantly higher expressed in weak dormancy line than that in nondormancy line. And the significant effects of *BnaC01T0313900ZS* and *BnaC03T0300500ZS* variation on seed dormancy were also demonstrated in present study. These findings provide a key information for investigating the function of *BnaSDR*s on seed dormancy in *B. napus*.

**Supplementary Information:**

The online version contains supplementary material available at 10.1186/s12870-023-04700-2.

## Introduction

Dormancy has lost in most crops with the long-term domestication [1]. As a consequence, pre-harvest sprouting (PHS), seeds tend to sprout or even germinate on field plants during continuous rainy weather before harvest, occurring on a large scale [2, 3]. And PHS has caused serious yield loss of many crops in China [4]. For decreasing yield loss, the genetic mechanisms of seed dormancy and PHS need to be explored in crops.

Among a series of reported seed dormancy QTLs, the functions of short-chain dehydrogenase/reductase 1 (*SDR*1) and *SDR*4 regulating rice seed dormancy have been clarified [5, 6]. *OsSDR*4, encodes a nucleus located protein, is the dominant genetic factor for the difference in pre-harvest sprouting between japonica and indica rice subspecies [7]. The *OsSDR*1 encodes an inhibitory protein that acts as a molecular link between the nutritional signaling pathway and plant hormone biosynthesis. And *OsSDR1* gene could regulate seed dormancy by interacting with other structural elements [8].

*SDRs*, one of the oldest and largest superfamily, can regulate growth and development in various species such as archaea, prokaryotes, eukaryotes and viruses [9, 10]. *ABA2/GIN1* (*AtSDR1*), the first identified *SDR* gene in *Arabidopsis*, was involved in sugar signaling and ABA biosynthesis. The loss-of-function mutation *Atsdr1* leads to a typical ABA-deficient phenotype in *Arabidopsis*, resulting in reduced seed dormancy. In contrast, overexpressing *AtSDR1 Arabidopsis* showed increased ABA content, promoted seed dormancy [11]. Thus, *SDR* genes have played critical role in seed dormancy in plant.

Rapeseed (*Brassica napus L.*) originated from natural hybridization between *B. rapa* and *B. oleracea* > 7,500 years ago [12]. As a young crop, it is an ideal model for seed dormancy investigation. As statistics in recent years, the proportion of rapeseed PHS in the main production area Yangtze River Basin of China was higher than 0.6%. And PHS has been seriously decrease seed weight and oil content in *B. napus* [13]. However, few report of seed dormancy and PHS mechanism has greatly inhibited the rapeseed breeding progress. It is great significance to clarify whether *SDR* genes are involved in seed dormancy and PHS in *B. napus*.

Considering these matters, 142 *BnaSDRs* were identified in *B. napus* genome by homology sequence blast in this study. The protein physicochemical properties, conserved motif, gene structure, cis-acting element, and tissue expression profile analysis were also conducted. Furthermore, seven seed dormancy related candidate genes were selected for gene expression and variation analysis. These findings could provide a key information for investigating the function of *BnaSDRs* on seed dormancy and PHS in *B. napus*.

## Materials and methods

### Identification of *SDR* genes in *B. napus* genome

Fifty-five AtSDR protein sequences obtained from UniProt database [14] (https://www.uniprot.org/) were employed as reference sequence in present study. Then, blastP [15] were performed in the *Brassica napus* pan-genome Information Resource (BnPIR) database [16] (http://cbi.hzau.edu.cn/bnapus/). BnaSDRs were screened according to the conserved protein structure by using the Simple Modular Architecture Research Tool (SMART) [17]. Besides, NCBI and Ensemble Plants data source were used to further validate the identified proteins.

The *B. napus* genome file, chromosome annotation information, and *BnaSDR* genes’ location information were downloaded from the BnPIR database. And Gene Location Visualize subroutine of TBtools software [18] was used to map the chromosome location of *BnaSDR* genes.

### Evolutionary analysis of SDR family

The full-length protein sequences of 55 AtSDRs, 142 BnaSDRs, 70 BraSDRs and 51 BolSDRs were obtained from UniProt database and BnPIR database, respectively. And these protein sequences were aligned using the ClustalW program [19]. Neighbor-Joining (NJ) phylogenetic tree was constructed by MEGA7.0 [20]. Bootstrap analysis was conducted with 1000 replications [21]. Then the evolutionary tree was visualized by using the online website iTOL (https://itol.embl.de/).

### Analysis of collinearity replication relationship of *BnaSDR* genes

In order to analyze the replication events involved in *BnaSDR* genes between or within species, the genome and annotation files of *Arabidopsis thaliana* and *B. napus* were downloaded from the Ensemble Plants database and BnPIR database, respectively. The OneStepMCScanX program in TBtools was used to analyze the collinearity between or within species of *BnaSDR* genes.

### Protein physicochemical properties and subcellular localization analysis of BnaSDR proteins

The physicochemical properties including amino acid number, molecular weight, theoretical pI, instability index, aliphatic index and grand average of hydropathicity of SDR proteins were analyzed by online software Expasy [22] (https://web.expasy.org/protparam/). Subcellular localization of SDR proteins were predicted by the online software Wolf PSORT [23] (https://wolfpsort.hgc.jp/).

### Analysis of conserved motifs of SDR proteins

The online analysis software MEME [24] (https://meme-suite.org/meme/) was used to analyze the conserved motifs of all SDR protein sequences, with the following parameters: distribution of motifs, the optimum width of motif, 6–50; the number of repetitions, any. number of motifs, 12; the number of motif occurrences on each sequence is not limited; Then visualization was conducted by TBtools software.

### Analysis of gene structure and cis-acting elements in promoter region of *SDR* genes

The rapeseed genome and annotation files were downloaded from the BnPIR database. And the *BnaSDR* gene structure was visualized by the subroutine Gene Structure View function of TBtools software. For cis-acting elements analysis, the upstream 1500 bp promoter sequence of *BnaSDR* genes were extracted from *B. napus* genome by TBtools software. Then, cis-acting elements in the *BnaSDR* promoter regions were predicted by online software Plant CARE [25] (http://bioinformatics.psb.ugent.be/webtools/plantcare/html/).

### Analysis of tissue expression profiles of *BnaSDR* genes

In order to investigate the tissue expression profiles of *BnaSDR* genes, the gene expression data in root, stem, leaf, bud, filament, petal, pollen, sepal, cotyledon, seed and silique of *B. napus* were obtained from BnTIR database [26] (http://yanglab.hzau.edu.cn). Considering *BnaSDR* might relate to seed dormancy, *BnaSDR* expression level in seeds from 14 to 64 days after flowering were also downloaded from BnTIR database.

### RNA extraction and qRT-PCR analysis

Primers were designed using qPrimerDB [27], and the specific primer sequence was shown in Table 1. The fresh harvest seeds of weak dormancy line (T) and nondormancy line (S) divided from a breeding population of our research group were employed for germination test. Seeds of these two lines were kept in rapeseed laboratory of Anhui agricultural university. The total RNA of fresh harvest seeds and soaked with ABA or water for 12 h of T or S line were isolated using plant total RNA extraction Kit (Baori Medical Technology (Beijing) Co., Ltd.,). The PrimeScriptTM RT reagent Kit with gDNA Eraser reverse transcription Kit (Baori Medical Technology (Beijing) Co., Ltd.,) was used to synthesize single-stranded cDNA. The qRT-PCR was conducted by real-time fluorescence quantitative PCR Kit (Baori Medical Technology (Beijing) Co., Ltd.,). The expression level of *BnaSDR* genes was quantified by 2^-△Ct^ method. Each test was repeated for four times. The expression heat map was drawn by TBtools software.

**Table 1:** Sequences of primers used for qRT-PCR

Gene	Forward primer	Reverse primer
*BnaA01T0256500ZS*	CTTGCTTGTAACCATTGCTCAT	CCATTGTCGTTTTAAGACGGTC
*BnaC01T0313900ZS*	TAGAGCCGTGAGTTACTGTTTT	CATTTGTAGAGTCTCTTGTGCG
*BnaA05T0476100ZS*	TTCCATTGATGAGATCGAGGAG	CAATAGCTCCTTTTGTAGACGC
*BnaA02T0152200ZS*	GGTTGCAGATTGATGTTCTTGT	GTATCAACCTCACTCTACTCCG
*BnaA03T0253100ZS*	GGAATGTCTATCCGACGTATGT	CCATTTAGCTCGAATCTCAACG
*BnaC03T0300500ZS*	CTCCTCTGTATACCGAAAGTCC	TTAAAACGTGAAAACCGAGGTG
*BnaC03T0290800ZS*	TGGTCGTTGAGTGTCAATATCA	GAAGAAAGGTGCAGTAAGGTTG
*Actin*	AACCTTCTCTCAAGTCTCTGTG	CCAGAATCATCACAAAGCATCC

### *BnaSDR* haplotype variation analysis

In order to analyze the effects of *BnaSDR* gene variation on seed dormancy, the variation of seven seed dormancy related candidate genes and germination character were investigated in a 143 rapeseed micro-core collections. The sequence variation and haplotype information were extracted from our previous study [12]. In germination experiment, fresh harvest-seeds of 143 rapeseed accessions were employed. Fifty seeds of each accession were sown on the plastic dish with one layer of filter paper. Seeds were germinated at 25 °C, a relative humidity of 60–70%, a 16-h photoperiod, in a growth chamber. Then, the germination rate was measured every day. All the treatments were replicated for three times.

## Results

### Identification of *SDR* genes in *B. napus* genome

**Fig. 1:**
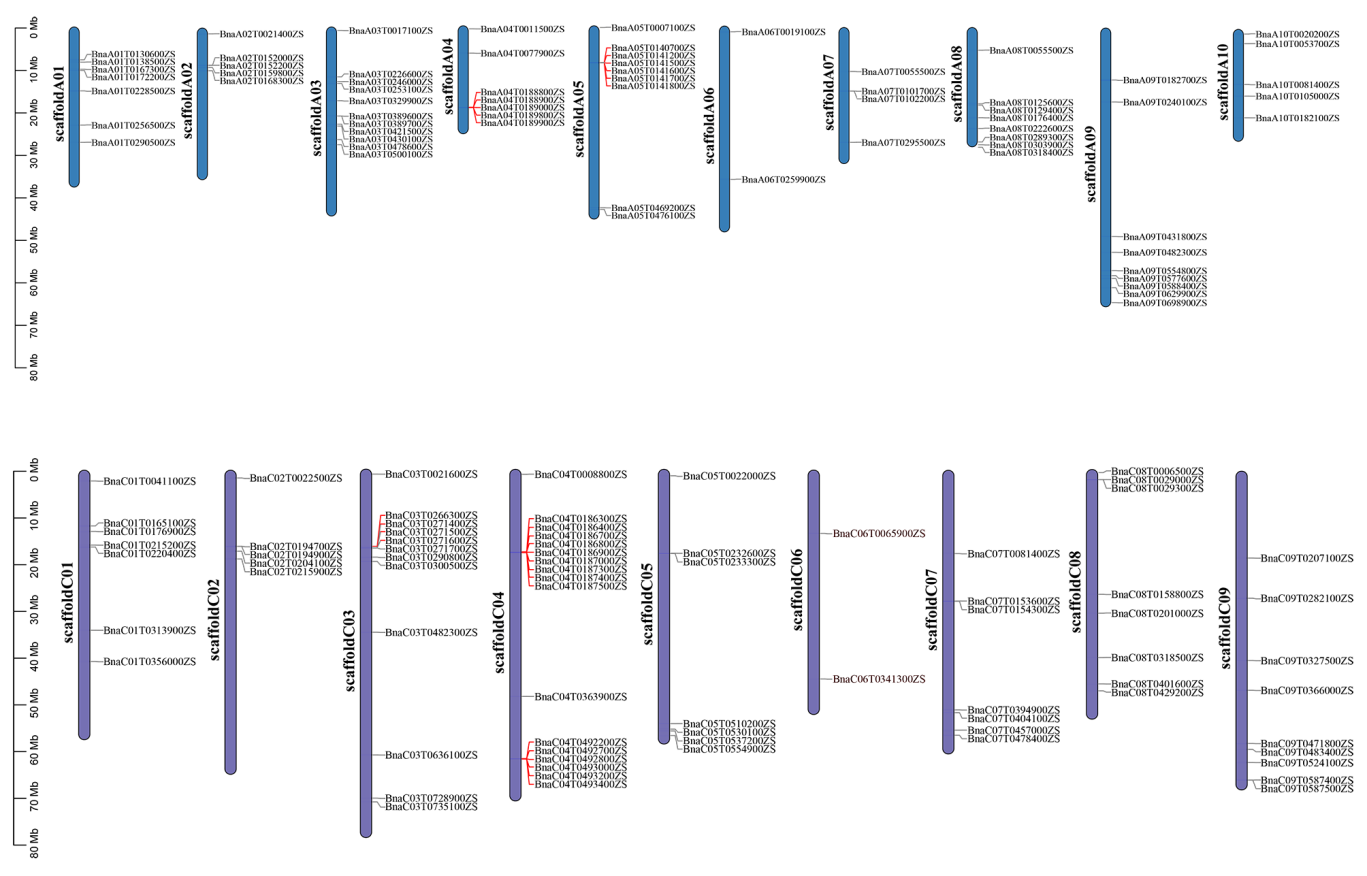
The distribution of *BnaSDR* genes in the *B. napus* chromosomes | *BnaSDR* gene clusters were marker as red line

A total of 142 *BnaSDR* genes were identified from *B. napus* genome by blast with 55 *AtSDR* sequences. The results of chromosome location shown that there were 141 *Bna*SDR genes anchored in 19 *B. napus* chromosomes (Fig. 1). However, *Bnascaffold2694G0000200ZS* has not been anchored to any chromosome. We also found that 141 *BnaSDR* genes were unevenly distributed on the A and C sub-genomes. There were 67 and 74 *BnaSDR* genes located on A and C sub-genomes, respectively. Among them, chromosome C04 contained the maximum number of *BnaSDRs* (17), and chromosome A06 and C06 had only two *BnaSDRs*. Furthermore, four *BnaSDR* gene clusters, contain more than five *BnaSDR*s in a 20 M region, were identified on chromosomes A04, A05, C03 and C04 (Fig. 1).

### Evolution of SDR family in *B. napus*

**Fig. 2:**
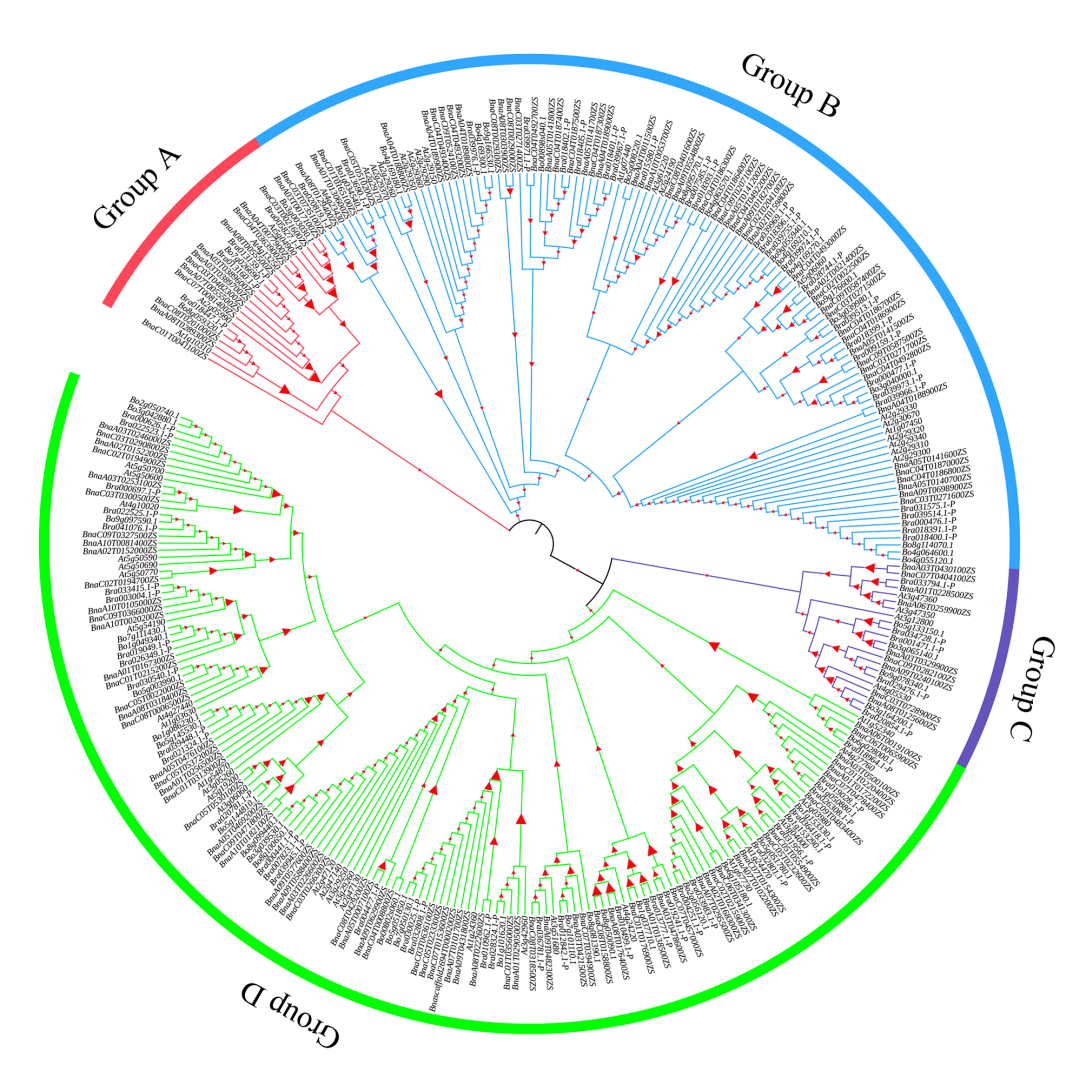
Phylogenetic tree of BnaSDR, BraSDR, BolSDR and AtSDR proteins

To understand the evolutionary relationships and functions among BnaSDR proteins, a phylogenetic tree was created with 142 BnaSDR protein sequences, its orthologous proteins in *B. rapa* and *B. oleracea*, and 55 AtSDR protein sequences by MEGA7.0. Phylogenetic tree results shown that 142 BnaSDR proteins were divided into four distinguishing groups (Group A–D; Fig. 2). The subgroup C contained the least number of SDR members, only 4 AtSDRs, 9 BnaSDRs, 5 BraSDRs and 4 BolSDRs. But Subgroup D, a largest group, contains 28 AtSDRs, 68 BnaSDRs, 34 BraSDRs and 27 BolSDRs. Subgroup A contained 4 AtSDRs, 13 BnaSDRs, 4 BraSDRs and 3 BolSDRs. Subgroup B contained 19 AtSDRs, 52 BnaSDRs, 27 BraSDRs and 17 BolSDRs.

**Fig. 3:**
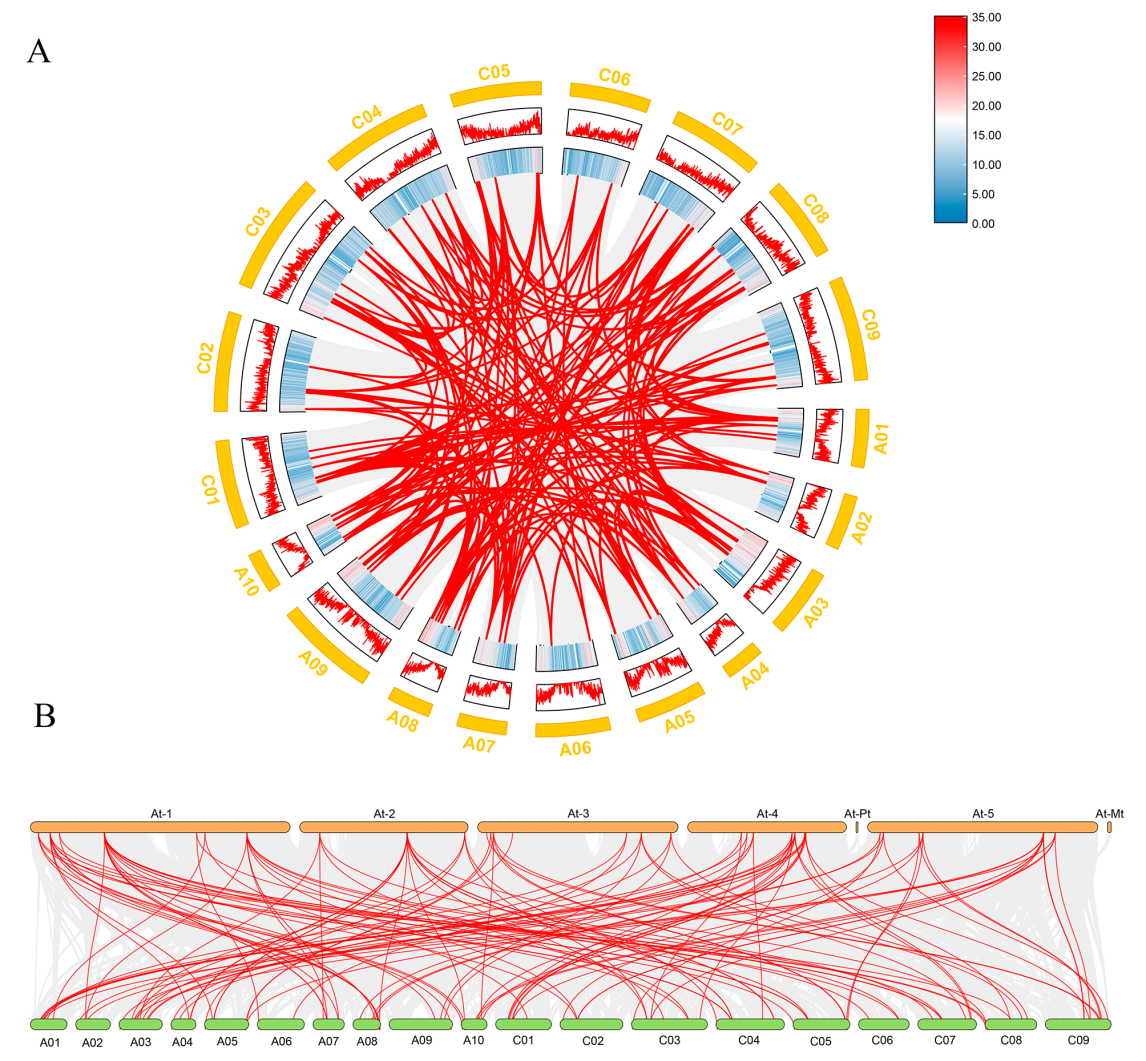
Synteny analysis of SDRs among *A. thaliana* and *B. napus* (**A**) Intraspecies collinearity analysis; (**B**) Interspecies collinearity analysis. The coarse red line in the circle is the collinearity gene of *SDR* genes among species, and the fine gray line indicates all the collinearity genes contained among species

Gene replication plays an important role in the evolution of organisms, replicated genes lay the foundation for the physiological and morphological changes of individual plants [28]. In order to study the replication events of the *BnaSDR* gene family in the *B.napus* genome and the interspecies of *A. thaliana*, the interspecies and intraspecies collinearity of *A. thaliana* and *B.napus* were investigated by using TBtools software (Fig. 3). Results shown that there were a large number of orthologous *SDR* genes between *B. napus* and *A. thaliana*. Within *B. napus* genome, the *SDR* gene has been amplified to a certain extent. These indicating that gene replication plays a very important role in the evolutionary process of *B. napus*.

### Physicochemical properties of BnaSDR proteins

**Fig. 4:**
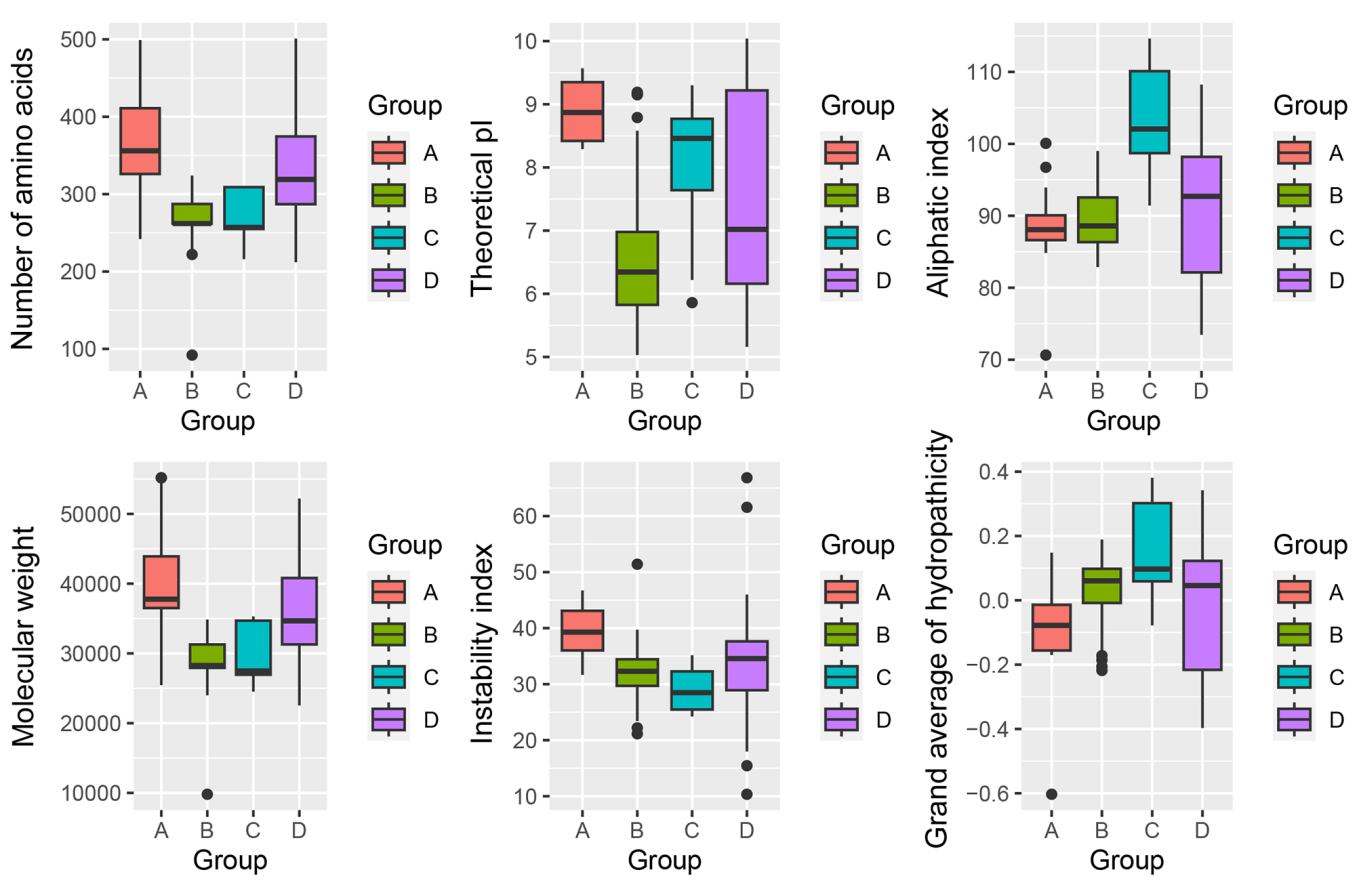
Comparations of protein physicochemical properties between different BnaSDR subgroups

In order to clarify the basic characteristics of SDR proteins in *B. napus*, the physicochemical properties of 142 BnaSDR proteins were analyzed. Results shown that the sequence length of BnaSDR proteins were ranged from 92 to 501 aa, with an average length of 306 aa. The largest relative molecular mass was BnaA04T0077900ZS (55188.59 Da), the smallest relative molecular mass was BnaC05T0510200ZS (9801.25 Da), and the average relative molecular mass was 33170.24 Da. The average theoretical pI was 7.37, and most BnaSDR proteins were alkaline. The instability index of BnaSDR proteins were between 10.35 and 66.84, and only 13.38% BnaSDR proteins were unstable proteins (Table S1). The average hydrophobicity index of BnaSDR proteins was between − 0.603 and 0.381, and 61.27% BnaSDR proteins showed hydrophobicity (Table S2; Fig. 4). Results of subcellular localization showed that most of the BnaSDR proteins (71, 50%) were located in chloroplasts, 33.10% BnaSDR proteins located in cytoplasm, and some BnaSDRs were located in peroxisomes (3.52%), plasma membrane (3.52%), nucleus (2.82%), endoplasmic reticulum (2.82%), cytoskeleton (2.11%) and extracellular matrix (1.41%). Only one BnaSDR protein (BnaC04T0186800ZS), homologous to At2g29330, was located on the Golgi apparatus (Table S1).

By comparing the physicochemical properties between different subgroups (Fig. 4), it was found that the number of amino acids, theoretical pI, molecular weight and instability index of subgroup A were the highest among four subgroups. And the unstable proteins were mainly distributed in subgroup A. The average theoretical pI of subgroup A and subgroup C was greater than 7. This indicated that most of the BnaSDRs in subgroup A and subgroup C were alkaline. BnaSDRs in subgroup D were mostly neutral. BnaSDRs in subgroup B were mostly acidic. The grand average of hydrophobicity of subgroup C was the highest. In addition, the BnaSDRs in B, C and D subgroups were almost hydrophobic proteins. However, BnaSDRs in subgroup A were hydrophilic proteins.

### Conserved motifs of BnaSDR proteins

**Fig. 5:**
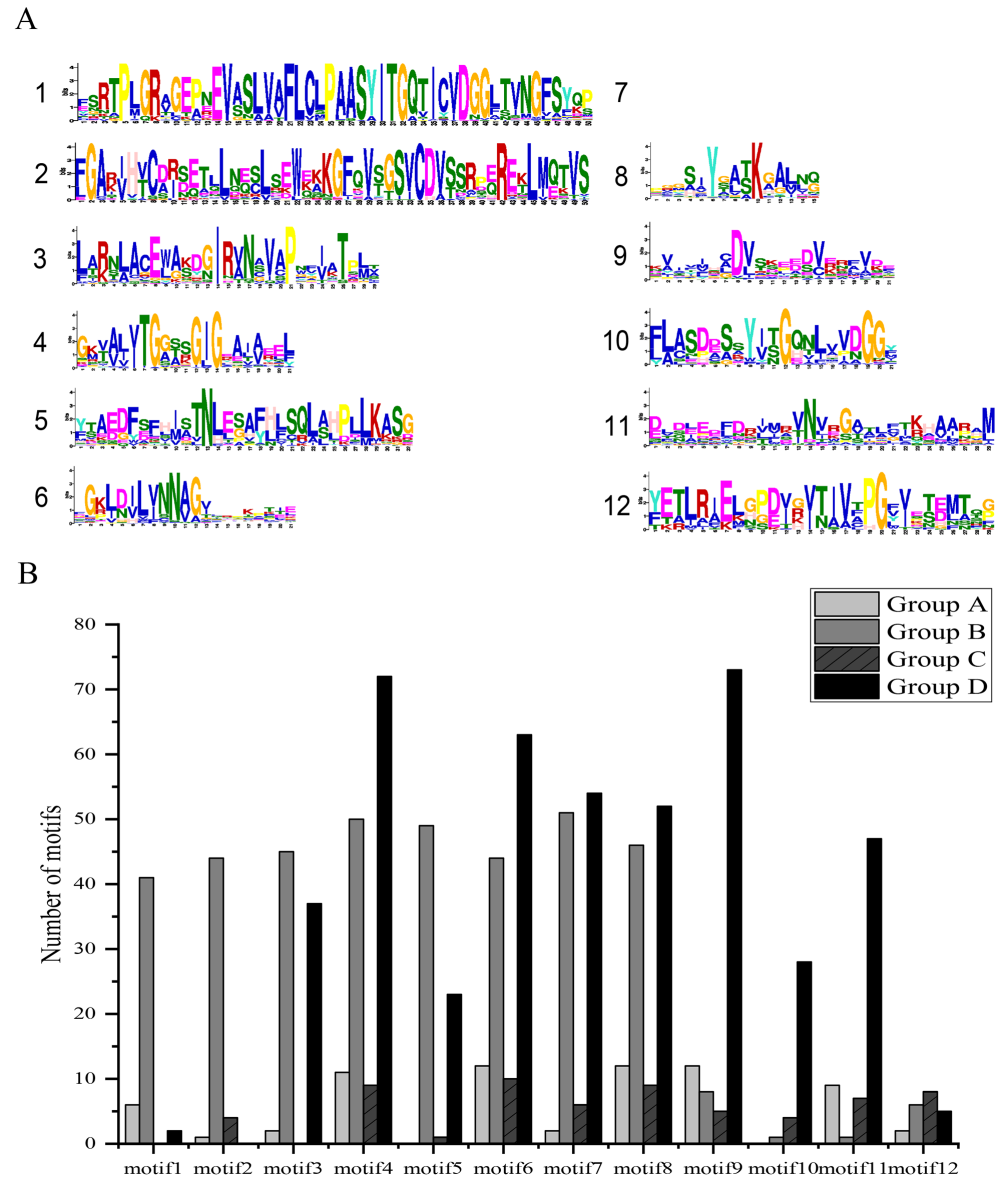
Motif distribution map of BnaSDRs. | (**A**) Conserved motifs in BnaSDRs; (**B**) Distribution of conserved motifs in different BnaSDR subgroups

To further understand the protein sequence features of BnaSDRs, the conserved motifs of each protein were identified using the online software MEME. we found that almost all of the BnaSDR proteins contained motif 4 (95.77%), indicating that motif 4 is relatively conserved in the SDR family. However, only 14.79% BnaSDR proteins contained the motif 12 (Fig. 5A, Figure S1).

Results of the distribution of conserved motifs in different groups have shown that proteins in the same group have similar motif distribution. It was found that motif 1 and motif 2 were mainly existed in the members of subgroup B. BnaSDRs containing motif 9, motif 10 and motif 11 are mainly in subgroup D (Fig. 5B). These indicating that the proteins in different subgroups may have large functional differentiation.

### Gene structure and cis-acting elements of *BnaSDR* genes

To character structures of *BnaSDR* genes, gene length, CDS length, intron number, and cis-acting elements were investigated. By aligning CDS and genomic sequences, we found that the structure and intron number were quite different among *BnaSDR* genes, but roughly similar among a subgroup. There were 2 to 12 introns distributed in *BnaSDR* genes. However, the number and location of introns in *BnaA09T0182700ZS* was very different from other branches in the same subgroup (Figure S2). The functional differentiation of *BnaA09T0182700ZS* has occurred in *B. napus*.

To explore cis-acting elements in the promoter region of *BnaSDR* genes, the online promoter database Plant Care was employed in present study. Results shown that there were 1558 (14 no duplication) cis-acting elements in the *BnaSDR* promoter regions. There were 856 hormone response regulatory elements (5 no duplication), including auxin-responsive element, gibberellin-responsive element, abscisic acid responsiveness, salicylic acid responsiveness and CGTCA-motif responsive element. There were 42.99% and 36.10% *BnaSDR* genes containing CGTCA-motif response element and abscisic acid response element, respectively (Table 2, Figure S3). These indicated that the expression of these genes might be regulated by hormone signaling.

Besides, there were 625 (5 no duplication) environmental stress responses elements, including light responsive element, anaerobic induction, defense and stress responsiveness, low-temperature responsiveness, MYB binding site involved in drought-inducibility. And the remaining 77 cis-elements (4 no duplication) are related to tissue expression, including meristem expression, MYB binding site involved in flavonoid biosynthetic genes regulation, endosperm expression and phytochrome down-regulation expression (Table 2, Figure S3). These results indicated that the functional diversity of *BnaSDR*s has been occured during the growth and development of *B. napus*.

**Table 2:** Distribution cis-acting elements in different *BnaSDR* subgroups

Groups		Group A	Group B	Group C	Group D	Total
Phytohormones responsive	Auxin	8	19	7	20	54
	GA	4	20	2	34	60
	ABA	34	112	19	144	309
	SA	7	21	7	30	65
	MeJA	36	146	26	160	368
Environmental responsive	Light	16	56	6	88	166
	Anaerobic	28	115	15	93	251
	Defense/stress	2	22	4	32	60
	Low-T	10	31	6	25	72
	Drought	3	38	5	30	76
Other responsive	Meristem	7	24	2	19	52
	Endosperm	0	5	1	8	14
	Phytochrome	0	0	0	2	2
	Flavonoid	1	5	0	3	9

### Tissue expression profiles of *BnaSDR* genes

To explore tissue-specific expression profiles of the *BnaSDR* genes, the expression data of 37 tissues (including 11 tissues and 26 seeds at the different development stages) were obtained from the BnTIR database. Results shown that the 142 *BnaSDR* genes widely expressed in different tissues (Figure S4). But the expression pattern of *BnaSDR* genes in different groups were largely different. The expression level in stem, leaf, bud and cotyledon of genes in subgroup A were higher than those in other groups. *BnaSDR* genes in subgroup C were highly expressed in root, filament, petal, pollen, sepal and silique, but not in cotyledon. In subgroup D, *BnaA02T0152200ZS*, *BnaC02T0194900ZS*, *BnaA03T0253100ZS* and *BnaC03T0300500ZS* were only highly expressed in seeds.

**Fig. 6:**
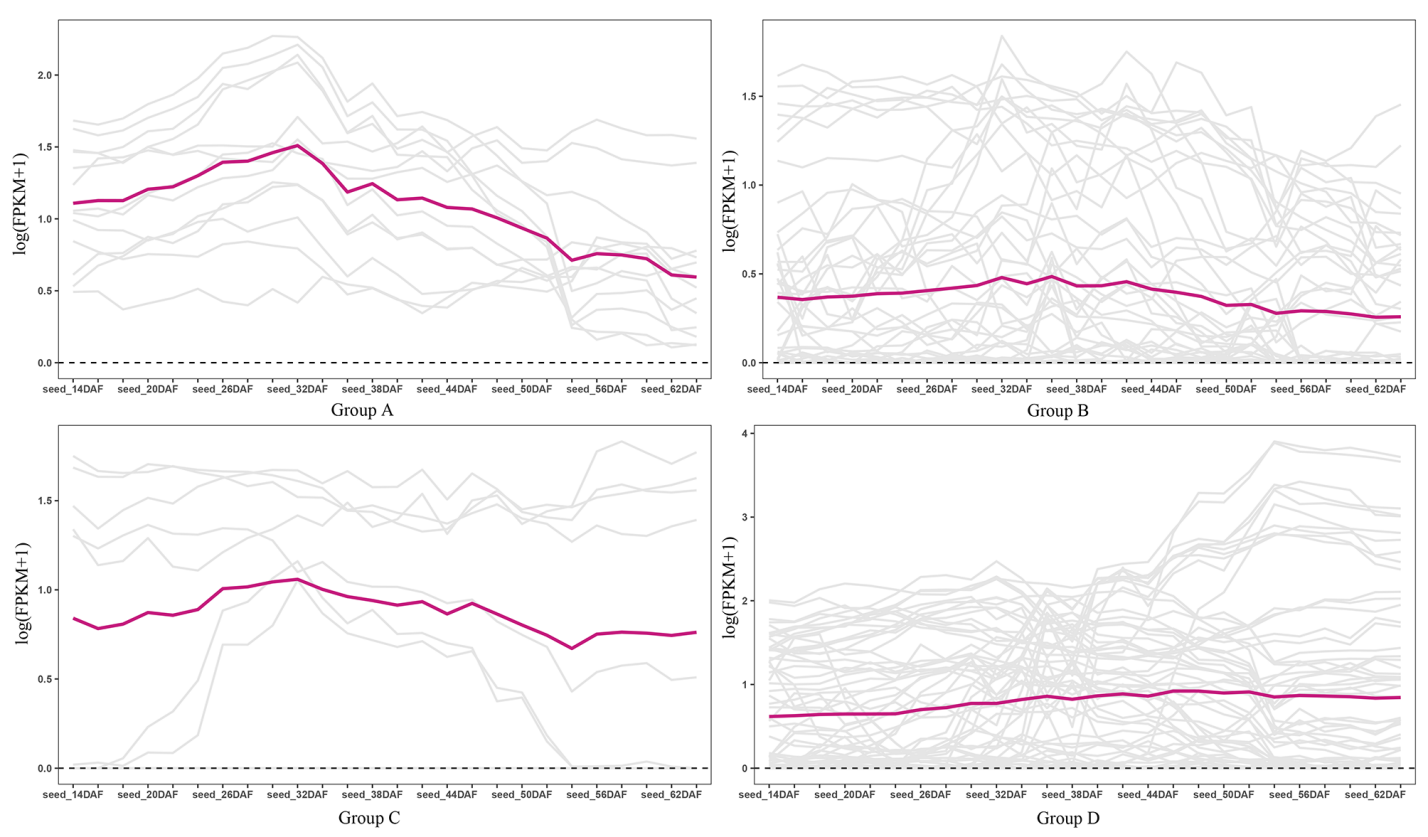
Expression patterns of *BnaSDR* gene in developing seeds

The expression level of *BnaSDR* genes in seeds from 14 to 64 days after flowering were also compared between different subgroups (Fig. 6). As a whole, the expression levels of *BnaSDR* genes in subgroup A, B and C were gradually decreased, but increased in subgroup D with seed development. Especially, the expression level of *BnaA01T0256500ZS*, *BnaC01T0313900ZS*, *BnaA05T0476100ZS, BnaA02T0152200ZS*, *BnaA03T0253100ZS*, *BnaC03T0300500ZS* and *BnaC03T0290800ZS* in subgroup D were significantly increased at the late stage of seed development (64 days after flowering).

### *BnaSDR* genes expression level in dormant seed

**Fig. 7:**
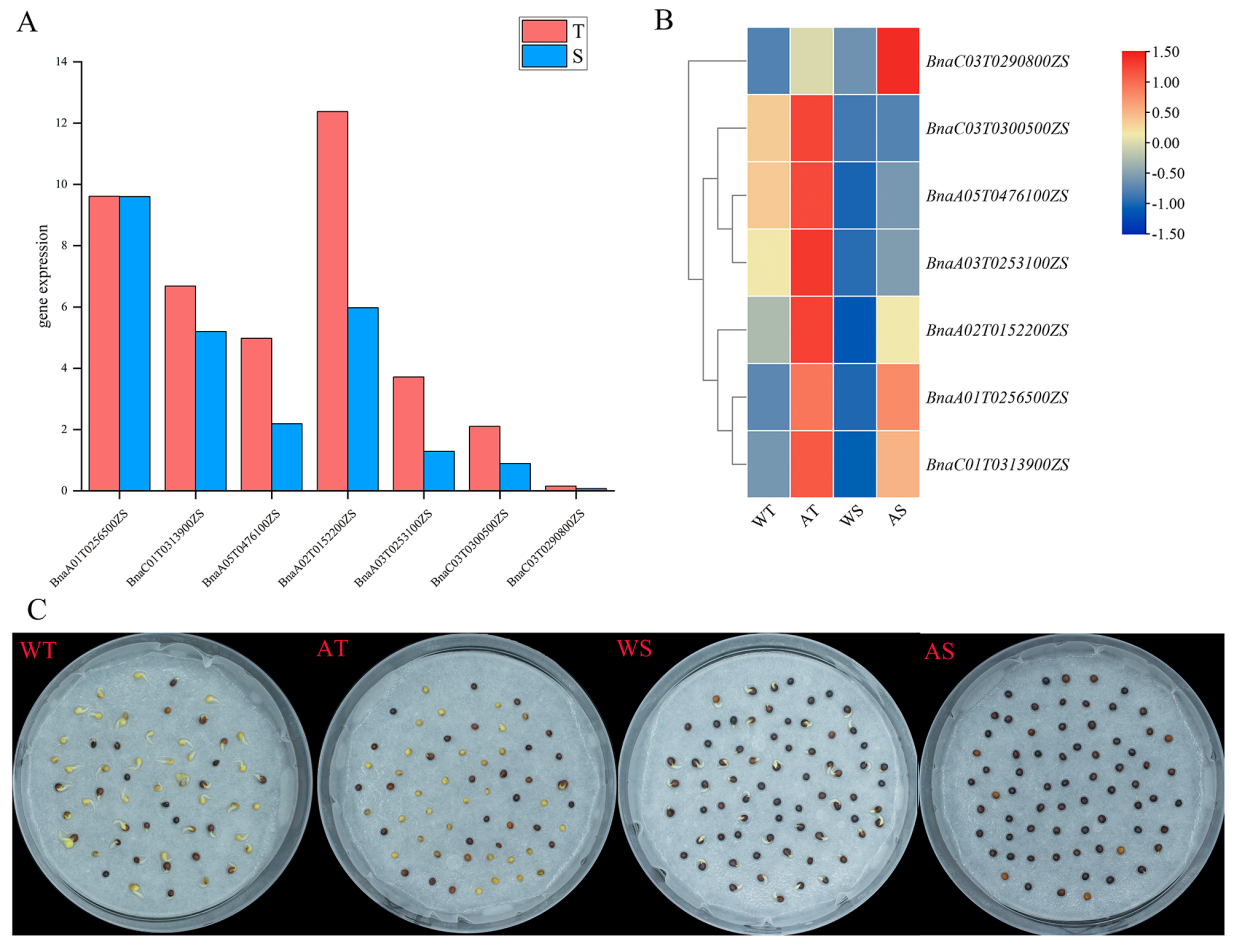
Expression pattern analysis of seven promising seed dormancy related *BnaSDR* genes. | (**A**) Expression level of seven promising seed dormancy related *BnaSDR* genes in fresh harvest seeds of weak dormant line (T) and non-dormant line (S); (**B**) Expression level of seven promising seed dormancy related *BnaSDR* genes in germinated seeds treated with distilled water or ABA for 12 h. (**C**) Phenotype of fresh harvest seeds treated with distilled water or ABA for 12 h. WT: fresh weak dormant seeds treated with distilled water for 12 h; WS: fresh non-dormant seeds treated with distilled water for 12 h; AT: fresh weak dormant seeds treated with ABA for 12 h; AS: fresh non-dormant seeds treated with ABA for 12 h

Based on the phylogenetic and gene expression data, seven promising seed dormancy related *BnaSDR* genes were selected from subgroup D for further study. Expression level of these seven genes in weak dormancy (T) or nondormancy (S) seeds were analyzed by qRT-PCR. Results shown that the expression levels of six *BnaSDRs* (*BnaC01T0313900ZS*, *BnaA05T0476100ZS*, *BnaA02T0152200ZS*, *BnaA03T0253100ZS*, *BnaC03T0300500ZS*, *BnaC03T0290800ZS*) in fresh harvest seeds of T line were significantly higher than those in S line (Fig. 7A). These indicating that *BnaC01T0313900ZS, BnaA05T0476100ZS, BnaA02T0152200ZS, BnaA03T0253100ZS, BnaC03T0300500ZS, BnaC03T0290800ZS* might relate to seed dormancy.

By comparing the gene expression levels of germinated seeds treated with distilled water or ABA, we found that abscisic acid treatment significantly increased the expression levels of seven genes both in T and S lines (Fig. 7B). The delayed germination was also found under ABA treatment (Fig. 7C). Thus, ABA treatment might enhance seed dormancy by upregulating seed dormancy related *BnaSDR* genes in *B napus*.

### Effects of *BnaSDR* variation on seed dormancy

In order to clarify the effects of *BnaSDR* gene variation on seed dormancy, the variation of seven seed dormancy related candidate genes and germination character were investigated in a 143 rapeseed micro-core collections. Results of sequence variation were shown that there were 1 to 9 variation sites in four *BnaSDR* genes (*BnaC01T0313900ZS*, *BnaC03T0300500ZS*, *BnaA02T0152200ZS*, and *BnaA03T0253100ZS*) (Table S3). However no available variation (minor allele frequency > 0.05) be detected of other three candidate genes in present population.

**Fig. 8:**
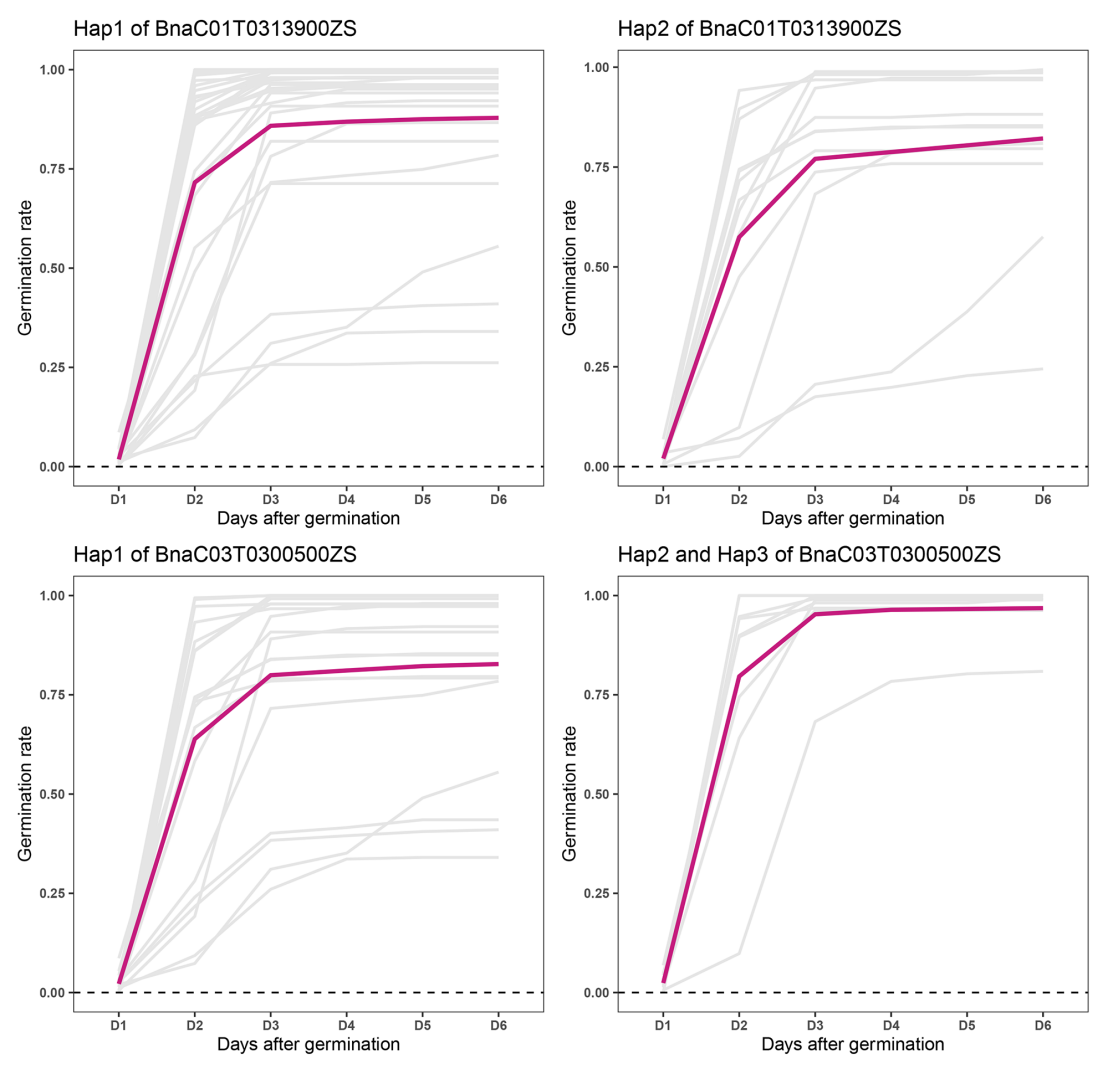
Effects of *BnaSDR* gene variation on seed dormancy in *B. napus*

There was one variation site in *BnaC01T0313900ZS*. Accessions carrying haplotypes 1 (Hap1, T variation) exhibited a higher germination speed and germination rate than that of Hap2 (C variation) (Fig. 8). And we also found that the germination speed and germination rate were significantly different between three haplotypes of *BnaC03T0300500ZS*. Accessions carrying Hap2 or Hap3 of *BnaC03T0300500ZS* exhibited higher germination speed and germination rate than that of Hap1 of *BnaC03T0300500ZS* (Fig. 8). However, there were no difference of germination character between different haplotypes in *BnaA02T0152200ZS* and *BnaA03T0253100ZS* (Figure S5).

## Discussions

Seed dormancy and its related PHS are complex quantitative traits controlled by multiple genes [29]. It has been demonstrated that *SDR* genes can regulate seed dormancy and PHS by promoting ABA biosynthesis [7]. In present study, a total of 142 non-repetitive *BnaSDR* genes were identified in the *B. napus* genome. Which provide a chance to clarify whether *SDR* genes are the key factor of seed dormancy in *B. napus*. The distribution of these 142 *BnaSDR* genes on *B. napus* chromosomes was irregular. And four *BnaSDR* gene clusters were found on chromosomes A04, A05, C03 and C04. As reported in previous studies, gene clusters may emerge on chromosomes when genes replicate. And it plays a special role in rapid stress response, gene coregulation, co-function and co-heredity, they are important for plant environmental adaptation and evolution [30, 31]. Therefore, it is speculated that *BnaSDR* genes were asymmetrically retained on these chromosomes after whole-genome triplication (WGT) event in *Brassica* [32]. These indicated *BnaSDR* genes play vital role in rapeseed growth and development.

We also found that the *SDR* family can be divided into four groups by phylogenetic analysis. The seed dormancy related protein ABA2 (At1g52340) [11] was distributed in the subgroup D. Members of same group have a similar protein character, conserved motifs, gene structure, cis-acting elements and tissue expression profile. There were also some homologous genes with different motifs and gene struct. These indicating that these genes in different subgroups may have large functional differentiation. Previous studies have reported that, *SDR* genes also involved in the synthesis and degradation of lipids, sugars, nucleotides and amino acids to regulate plant growth, development and stress defense [33, 34].

The expression pattern plays an important role in gene function [35]. We found that the expression level of seven *BnaSDRs* (*BnaA01T0256500ZS*, *BnaC01T0313900ZS*, *BnaA05T0476100ZS, BnaA02T0152200ZS*, *BnaA03T0253100ZS*, *BnaC03T0300500ZS* and *BnaC03T0290800ZS)* in subgroup D were rapidly raised at the late stage of seed development (64 days after flowering). The expression of six *BnaSDRs* (*BnaC01T0313900ZS*, *BnaA05T0476100ZS*, *BnaA02T0152200ZS*, *BnaA03T0253100ZS*, *BnaC03T0300500ZS*, *BnaC03T0290800ZS*) among these seven genes in T line (weak dormancy) was higher than that in S line (non-dormancy). In addition, the expression levels were increased to varying degrees after ABA treatment in both T and S lines. Previous studies have shown that the multi-step reaction of *AtSDR1* catalyzing aflatoxin to abscisic aldehyde is a key step in ABA biosynthesis [36–38]. And the seed dormancy is enhanced during ABA biosynthesis. Thus, these six *BnaSDRs* might play an important role in rapeseed seed dormancy. Which provide a critical information for investigating the molecular mechanism of seed dormancy and PHS in *B. napus*.

The role of ABA and GAs on seed dormancy and germination have been demonstrated by previous studies [39–41]. In present study, a large number of hormone response-related elements and abiotic stress-related elements in the upstream promoter region of *BnaSDR* genes were also identified. And ABA treatment significantly increased the expression levels of seven seed dormancy related candidate genes both in T and S lines in present study. Therefore, *BnaSDR* genes might regulate seed dormancy as well as growth and development by responding hormone in *B. napus* as in other plants.

### Electronic supplementary material

Below is the link to the electronic supplementary material.

Supplementary Material 1

## Data Availability

The datasets used and/or analyzed during the current study are available from the corresponding author on reasonable request.
