# Supplementary material for: Genome-wide characterization of SDR gene family and its potential role in seed dormancy of Brassica napus L
Source: BMC Plant Biol. 2024 Jan 2;24:21. doi: 10.1186/s12870-023-04700-2 (PMC10759766; doi:10.1186/s12870-023-04700-2)
Supplement: Supplementary file 1 — Supplementary Material 1 [file 12870_2023_4700_MOESM1_ESM.docx]

**Supplementary Materials**

**Table S1 Physicochemical properties and subcellular localization of SDR gene family proteins**

| **Gene** | **Number of amino acids** | **Molecular weight** | **Theoretical pI** | **Instability index** | **Aliphatic index** | **Grand average of hydropathicity** | **subcellular localization** |
| --- | --- | --- | --- | --- | --- | --- | --- |
| BnaA04T0077900ZS | 499 | 55188.59 | 9.52 | 36.12 | 88.84 | -0.156 | cyto |
| BnaA08T0055500ZS | 497 | 54836.11 | 9.37 | 35.13 | 87.81 | -0.170 | nucl |
| BnaC04T0363900ZS | 499 | 55173.58 | 9.57 | 36.02 | 88.84 | -0.156 | cyto |
| BnaC08T0006500ZS | 398 | 43545.45 | 9.30 | 34.34 | 77.51 | -0.397 | chlo |
| BnaA08T0318400ZS | 398 | 43579.49 | 9.20 | 32.47 | 77.01 | -0.390 | chlo |
| BnaA10T0020200ZS | 400 | 43743.81 | 9.31 | 33.93 | 78.32 | -0.350 | chlo |
| BnaC05T0022000ZS | 400 | 43764.82 | 9.17 | 35.13 | 78.32 | -0.336 | chlo |
| BnaC02T0215900ZS | 319 | 35672.73 | 9.57 | 32.50 | 95.96 | 0.030 | chlo |
| BnaC06T0341300ZS | 237 | 26451.96 | 9.33 | 27.91 | 104.89 | 0.067 | chlo |
| BnaA02T0168300ZS | 319 | 35562.64 | 9.63 | 33.20 | 98.12 | 0.048 | chlo |
| BnaA07T0295500ZS | 318 | 35700.70 | 9.60 | 38.28 | 96.89 | -0.031 | chlo |
| BnaC01T0215200ZS | 397 | 42951.90 | 9.28 | 35.40 | 82.37 | -0.179 | chlo |
| BnaA01T0167300ZS | 397 | 42902.83 | 9.29 | 35.40 | 82.37 | -0.184 | chlo |
| BnaC09T0366000ZS | 349 | 38206.60 | 9.79 | 29.01 | 84.13 | -0.250 | chlo |
| BnaA10T0105000ZS | 404 | 43963.10 | 9.68 | 32.13 | 81.41 | -0.212 | chlo |
| BnaA04T0189900ZS | 279 | 29927.10 | 5.54 | 37.49 | 89.43 | 0.073 | chlo |
| BnaC04T0493400ZS | 297 | 32150.80 | 6.39 | 38.89 | 88.28 | 0.042 | cyto |
| BnaC01T0176900ZS | 389 | 44101.99 | 5.26 | 34.80 | 76.14 | -0.361 | cyto |
| BnaC07T0457000ZS | 380 | 42774.92 | 7.04 | 37.33 | 79.24 | -0.287 | cyto |
| BnaA01T0138500ZS | 390 | 44108.10 | 5.42 | 34.81 | 76.95 | -0.335 | cyto |
| BnaC08T0158800ZS | 390 | 44206.25 | 5.35 | 35.05 | 78.18 | -0.313 | cyto |
| BnaA03T0478600ZS | 379 | 42607.57 | 7.00 | 34.93 | 79.18 | -0.319 | cyto |
| BnaA08T0176400ZS | 395 | 44644.46 | 5.16 | 40.11 | 76.71 | -0.367 | cyto |
| BnaA06T0019100ZS | 284 | 29879.86 | 5.78 | 26.93 | 95.42 | 0.118 | cyto |
| BnaC06T0065900ZS | 282 | 29746.90 | 5.91 | 25.85 | 98.16 | 0.164 | cyto |
| BnaC07T0081400ZS | 411 | 43915.17 | 9.35 | 41.38 | 90.07 | -0.014 | chlo |
| BnaA07T0055500ZS | 276 | 29763.36 | 8.31 | 36.84 | 100.07 | 0.143 | cyto |
| BnaC03T0482300ZS | 356 | 37590.49 | 8.29 | 46.73 | 84.83 | -0.102 | chlo |
| BnaA03T0389700ZS | 394 | 41534.15 | 8.59 | 39.65 | 88.07 | -0.018 | chlo |
| BnaA03T0389600ZS | 379 | 39885.23 | 8.87 | 39.29 | 86.62 | -0.040 | chlo |
| BnaC03T0021600ZS | 347 | 37636.16 | 8.92 | 43.09 | 86.83 | -0.078 | chlo |
| BnaA03T0017100ZS | 347 | 37777.25 | 8.80 | 43.76 | 86.25 | -0.106 | chlo |
| BnaA01T0130600ZS | 324 | 34844.66 | 9.15 | 31.24 | 85.49 | -0.167 | chlo |
| BnaC01T0165100ZS | 322 | 34640.43 | 9.17 | 30.61 | 86.02 | -0.161 | chlo |
| BnaC03T0735100ZS | 322 | 34729.40 | 8.79 | 35.65 | 86.61 | -0.086 | chlo |
| BnaA08T0129400ZS | 322 | 34746.39 | 8.58 | 35.19 | 86.30 | -0.088 | chlo |
| BnaA08T0289300ZS | 242 | 25440.38 | 8.42 | 31.94 | 96.74 | 0.148 | chlo |
| BnaC08T0201000ZS | 242 | 25449.34 | 8.42 | 31.66 | 93.93 | 0.135 | chlo |
| BnaC02T0194900ZS | 349 | 39114.91 | 6.26 | 40.91 | 89.94 | 0.046 | cyto |
| BnaA02T0152200ZS | 349 | 39041.92 | 6.04 | 39.72 | 90.77 | 0.052 | cyto |
| BnaC03T0728900ZS | 258 | 27537.87 | 9.30 | 32.28 | 99.42 | 0.034 | pero |
| BnaA08T0125600ZS | 257 | 27478.74 | 8.80 | 31.37 | 102.06 | 0.059 | pero |
| BnaC09T0282100ZS | 255 | 26945.04 | 8.46 | 33.26 | 98.71 | 0.097 | pero |
| BnaA09T0240100ZS | 255 | 27065.17 | 7.75 | 35.16 | 97.18 | 0.065 | pero |
| BnaC05T0530100ZS | 324 | 34943.41 | 8.89 | 43.88 | 103.86 | 0.180 | chlo |
| BnaA05T0469200ZS | 324 | 35071.59 | 9.08 | 43.98 | 102.65 | 0.147 | chlo |
| BnaC09T0471800ZS | 264 | 27869.15 | 9.36 | 36.93 | 96.48 | 0.027 | chlo |
| BnaA10T0182100ZS | 276 | 30076.71 | 8.68 | 33.02 | 87.32 | -0.017 | cysk |
| BnaC03T0290800ZS | 341 | 38246.03 | 6.16 | 33.39 | 92.02 | 0.109 | chlo |
| BnaA03T0246000ZS | 341 | 38284.12 | 6.16 | 34.07 | 92.61 | 0.119 | chlo |
| BnaA09T0431800ZS | 320 | 33671.12 | 9.50 | 36.50 | 95.47 | 0.141 | chlo |
| BnaA07T0101700ZS | 321 | 33822.16 | 8.96 | 33.10 | 93.64 | 0.088 | chlo |
| Bnascaffold2694T0000200ZS | 320 | 33671.12 | 9.50 | 36.50 | 95.47 | 0.141 | chlo |
| BnaC07T0153600ZS | 326 | 34536.96 | 9.08 | 35.67 | 91.90 | 0.049 | chlo |
| BnaC05T0233300ZS | 328 | 34919.51 | 9.40 | 35.57 | 92.80 | 0.115 | chlo |
| BnaC03T0636100ZS | 380 | 40639.37 | 8.80 | 41.91 | 80.92 | -0.231 | chlo |
| BnaA08T0222600ZS | 290 | 31288.58 | 5.73 | 35.25 | 84.45 | -0.214 | cyto |
| BnaA03T0500100ZS | 366 | 40512.96 | 8.99 | 42.89 | 100.93 | -0.024 | chlo |
| BnaC01T0220400ZS | 342 | 38070.17 | 5.18 | 45.76 | 86.40 | -0.223 | nucl |
| BnaA01T0172200ZS | 373 | 41331.19 | 5.20 | 44.32 | 91.21 | -0.095 | nucl |
| BnaC07T0478400ZS | 354 | 39011.70 | 5.87 | 40.55 | 95.25 | -0.053 | cyto |
| BnaC05T0232600ZS | 236 | 26695.25 | 9.99 | 33.45 | 101.69 | -0.073 | chlo |
| BnaA07T0102200ZS | 305 | 34373.29 | 9.82 | 35.15 | 106.10 | 0.224 | chlo |
| BnaC07T0154300ZS | 304 | 34307.20 | 10.04 | 37.43 | 103.55 | 0.174 | chlo |
| BnaC01T0313900ZS | 287 | 31460.60 | 6.19 | 33.08 | 78.85 | -0.310 | cysk |
| BnaA01T0256500ZS | 287 | 31448.55 | 6.19 | 32.29 | 77.49 | -0.326 | cysk |
| BnaC08T0401600ZS | 296 | 32766.42 | 5.47 | 32.21 | 92.52 | -0.172 | cyto |
| BnaA09T0554800ZS | 296 | 32859.46 | 5.48 | 32.40 | 88.21 | -0.206 | cyto |
| BnaA04T0011500ZS | 297 | 33260.00 | 5.03 | 35.57 | 94.51 | -0.218 | cyto |
| BnaA05T0141600ZS | 262 | 27911.81 | 6.30 | 33.57 | 86.76 | 0.066 | E.R. |
| BnaC04T0187000ZS | 262 | 27922.82 | 6.20 | 34.12 | 88.59 | 0.090 | chlo |
| BnaC04T0186800ZS | 245 | 26341.97 | 5.95 | 36.52 | 87.96 | 0.058 | golg |
| BnaC05T0554900ZS | 248 | 25886.40 | 5.97 | 31.26 | 97.58 | 0.124 | chlo |
| BnaC03T0266300ZS | 501 | 52196.59 | 6.19 | 17.98 | 97.33 | 0.179 | chlo |
| BnaA03T0226600ZS | 212 | 22541.97 | 9.19 | 24.17 | 95.71 | 0.072 | cyto |
| BnaA09T0588400ZS | 258 | 26857.78 | 6.08 | 18.29 | 98.29 | 0.189 | cyto |
| BnaC05T0537200ZS | 319 | 35115.31 | 6.72 | 35.90 | 86.52 | -0.028 | cyto |
| BnaA05T0476100ZS | 288 | 31278.56 | 6.25 | 38.81 | 80.62 | -0.166 | cyto |
| BnaA02T0152000ZS | 311 | 34336.07 | 8.04 | 28.99 | 103.44 | 0.342 | chlo |
| BnaC02T0194700ZS | 314 | 34796.68 | 8.86 | 27.39 | 105.25 | 0.285 | chlo |
| BnaA01T0228500ZS | 216 | 24507.62 | 5.86 | 24.88 | 114.63 | 0.381 | chlo |
| BnaA10T0081400ZS | 299 | 33147.69 | 8.91 | 42.98 | 108.23 | 0.233 | chlo |
| BnaC09T0327500ZS | 299 | 33262.87 | 9.05 | 45.99 | 108.23 | 0.225 | chlo |
| BnaC03T0300500ZS | 463 | 51971.85 | 6.71 | 66.84 | 73.46 | -0.331 | E.R. |
| BnaA03T0253100ZS | 461 | 51611.45 | 6.84 | 61.57 | 73.99 | -0.305 | E.R. |
| BnaA06T0259900ZS | 310 | 35300.23 | 7.64 | 24.24 | 108.81 | 0.214 | chlo |
| BnaC07T0404100ZS | 309 | 34732.79 | 8.77 | 25.47 | 110.42 | 0.320 | plas |
| BnaA03T0430100ZS | 309 | 34692.75 | 8.77 | 27.53 | 110.10 | 0.302 | plas |
| BnaC04T0186400ZS | 262 | 27855.99 | 7.56 | 28.10 | 88.59 | 0.106 | chlo |
| BnaA05T0141200ZS | 262 | 27984.14 | 6.51 | 31.59 | 92.29 | 0.136 | chlo |
| BnaC04T0492200ZS | 263 | 28097.91 | 5.54 | 21.15 | 89.01 | 0.066 | E.R. |
| BnaC04T0493000ZS | 243 | 26674.97 | 9.19 | 23.42 | 93.09 | 0.016 | cyto |
| BnaA05T0140700ZS | 262 | 27916.99 | 6.94 | 30.78 | 95.00 | 0.124 | chlo |
| BnaC04T0186300ZS | 262 | 28093.12 | 6.07 | 32.48 | 91.18 | 0.098 | chlo |
| BnaC02T0022500ZS | 291 | 31425.58 | 5.17 | 28.12 | 91.07 | -0.012 | cyto |
| BnaC09T0587500ZS | 261 | 28168.78 | 5.65 | 34.67 | 84.44 | -0.056 | plas |
| BnaA02T0021400ZS | 263 | 28230.05 | 6.90 | 29.75 | 90.42 | -0.032 | chlo |
| BnaC09T0587400ZS | 262 | 28011.96 | 6.83 | 28.18 | 97.86 | 0.060 | plas |
| BnaA09T0629900ZS | 264 | 28089.22 | 7.21 | 25.08 | 100.80 | 0.138 | extr |
| BnaA05T0007100ZS | 257 | 26763.39 | 5.98 | 15.45 | 99.42 | 0.159 | cyto |
| BnaC04T0008800ZS | 262 | 27335.16 | 5.98 | 10.35 | 102.71 | 0.194 | cyto |
| BnaC07T0394900ZS | 300 | 31767.22 | 6.31 | 23.89 | 90.07 | 0.046 | cyto |
| BnaA09T0482300ZS | 296 | 31343.82 | 5.99 | 26.07 | 92.91 | 0.120 | cyto |
| BnaA03T0421500ZS | 300 | 31767.22 | 6.31 | 23.89 | 90.07 | 0.046 | cyto |
| BnaC08T0318500ZS | 296 | 31356.98 | 6.50 | 26.26 | 91.59 | 0.097 | cyto |
| BnaC08T0429200ZS | 261 | 27763.69 | 6.61 | 24.30 | 98.97 | 0.057 | cyto |
| BnaA09T0577600ZS | 290 | 31312.72 | 6.76 | 26.13 | 99.17 | 0.029 | chlo |
| BnaC03T0271400ZS | 274 | 29424.83 | 6.53 | 29.55 | 88.58 | 0.084 | cyto |
| BnaC08T0029000ZS | 262 | 28117.13 | 6.81 | 32.54 | 85.99 | 0.087 | chlo |
| BnaA08T0303900ZS | 262 | 28279.34 | 6.81 | 31.89 | 85.23 | 0.072 | cyto |
| BnaC08T0029300ZS | 262 | 28024.86 | 6.31 | 31.70 | 86.37 | 0.021 | chlo |
| BnaA09T0698900ZS | 262 | 28248.34 | 6.31 | 27.31 | 88.93 | 0.098 | chlo |
| BnaC09T0524100ZS | 297 | 32071.73 | 6.98 | 27.81 | 86.36 | 0.070 | cyto |
| BnaC04T0493200ZS | 297 | 32097.82 | 6.98 | 26.52 | 87.68 | 0.085 | cyto |
| BnaA04T0189800ZS | 297 | 32200.79 | 6.99 | 29.44 | 85.05 | 0.027 | cyto |
| BnaC03T0271500ZS | 262 | 28350.15 | 6.11 | 33.55 | 92.63 | 0.021 | pero |
| BnaA04T0188900ZS | 268 | 28656.70 | 6.43 | 29.84 | 95.00 | 0.161 | plas |
| BnaA05T0141700ZS | 250 | 26532.38 | 7.58 | 22.20 | 91.28 | 0.129 | cyto |
| BnaC04T0187300ZS | 249 | 26794.90 | 7.60 | 30.93 | 90.88 | 0.105 | chlo |
| BnaC05T0510200ZS | 92 | 9801.25 | 5.19 | 51.41 | 85.98 | 0.189 | chlo |
| BnaC04T0492700ZS | 262 | 27896.86 | 6.59 | 32.83 | 91.95 | 0.107 | extr |
| BnaA05T0141800ZS | 262 | 28008.79 | 5.27 | 25.95 | 89.35 | 0.054 | cyto |
| BnaC04T0187400ZS | 262 | 28085.79 | 5.26 | 31.01 | 86.72 | 0.014 | chlo |
| BnaA04T0189000ZS | 262 | 27951.88 | 5.96 | 32.38 | 97.52 | 0.156 | chlo |
| BnaC03T0271700ZS | 262 | 28339.25 | 6.09 | 30.33 | 94.50 | 0.084 | chlo |
| BnaC01T0041100ZS | 326 | 36503.37 | 9.23 | 44.78 | 70.64 | -0.603 | nucl |
| BnaC04T0187500ZS | 286 | 31228.51 | 6.10 | 35.94 | 82.87 | -0.186 | cyto |
| BnaC04T0186700ZS | 262 | 28442.55 | 5.85 | 34.35 | 97.14 | 0.084 | chlo |
| BnaC04T0186900ZS | 262 | 28294.43 | 6.11 | 33.28 | 99.01 | 0.128 | chlo |
| BnaC04T0492800ZS | 226 | 24002.51 | 7.60 | 32.57 | 96.28 | 0.118 | chlo |
| BnaA05T0141500ZS | 262 | 28332.29 | 5.75 | 38.56 | 98.28 | 0.098 | chlo |
| BnaA04T0188800ZS | 300 | 32793.28 | 8.45 | 35.18 | 86.37 | -0.125 | chlo |
| BnaA09T0182700ZS | 256 | 27166.80 | 5.64 | 28.33 | 84.30 | 0.061 | cyto |
| BnaC09T0207100ZS | 266 | 28348.04 | 5.52 | 36.27 | 84.40 | -0.002 | chlo |
| BnaC02T0204100ZS | 266 | 28454.22 | 5.97 | 33.70 | 86.20 | 0.025 | cyto |
| BnaA10T0053700ZS | 266 | 28323.28 | 6.74 | 30.06 | 86.20 | 0.054 | cyto |
| BnaA02T0159800ZS | 222 | 23994.19 | 6.38 | 34.36 | 88.38 | -0.030 | cyto |
| BnaC03T0271600ZS | 239 | 25958.83 | 8.47 | 39.72 | 95.48 | -0.007 | chlo |
| BnaC09T0483400ZS | 265 | 27894.82 | 7.69 | 38.57 | 92.45 | 0.101 | chlo |
| BnaA01T0290500ZS | 271 | 28313.38 | 6.39 | 28.71 | 95.39 | 0.122 | cyto |
| BnaC01T0356000ZS | 271 | 28351.43 | 6.71 | 29.91 | 94.32 | 0.095 | cyto |
| BnaA03T0329900ZS | 252 | 26851.61 | 6.22 | 28.49 | 91.43 | -0.078 | cyto |

Note: nucl: nucleus; cyto: cytoplasm; chlo: chloroplast; pero: peroxisome; cysk: cytoskeleton; E.R.: Endoplasmic reticulum; golg: Golgi apparatus; plas: plasma membrane; extr: extracell

**Table S2. Statistics on the physicochemical properties of BnaSDR gene family proteins**

|  | Max | MIN | Means | STEDV |
| --- | --- | --- | --- | --- |
| Number of amino acids | 501 | 92 | 306 | 63 |
| Molecular weight | 55188.59 | 9801.25 | 33170.24 | 7205.69 |
| Theoretical pI | 10.04 | 5.03 | 7.37 | 1.51 |
| Instability index | 66.84 | 10.35 | 33.48 | 7.33 |
| Aliphatic index | 114.63 | 70.64 | 91.24 | 8.34 |
| Grand average of hydropathicity | 0.381 | -0.603 | 0.001 | 0.170 |

**Table S3. Variation information of BnaSDR genes**

| Gene | Chromosome | Position | Alleles |
| --- | --- | --- | --- |
| BnaC03T0300500ZS | scaffoldC03 | 19725599 | A/G |
| BnaC03T0300500ZS | scaffoldC03 | 19725645 | T/A |
| BnaC03T0300500ZS | scaffoldC03 | 19725661 | C/T |
| BnaC03T0300500ZS | scaffoldC03 | 19726185 | G/T |
| BnaC03T0300500ZS | scaffoldC03 | 19726195 | C/T |
| BnaC03T0300500ZS | scaffoldC03 | 19726199 | G/A |
| BnaC03T0300500ZS | scaffoldC03 | 19726205 | A/C |
| BnaC01T0313900ZS | scaffoldC01 | 34243307 | T/C |
| BnaA03T0253100ZS | scaffoldA03 | 13273762 | A/T |
| BnaA03T0253100ZS | scaffoldA03 | 13273973 | T/C |
| BnaA03T0253100ZS | scaffoldA03 | 13274288 | A/G |
| BnaA03T0253100ZS | scaffoldA03 | 13274289 | T/C |
| BnaA03T0253100ZS | scaffoldA03 | 13274294 | A/G |
| BnaA03T0253100ZS | scaffoldA03 | 13274399 | C/T |
| BnaA03T0253100ZS | scaffoldA03 | 13274411 | G/A |
| BnaA03T0253100ZS | scaffoldA03 | 13274432 | A/T |
| BnaA03T0253100ZS | scaffoldA03 | 13274483 | T/G |
| BnaA02T0152200ZS | scaffoldA02 | 8763829 | C/T |


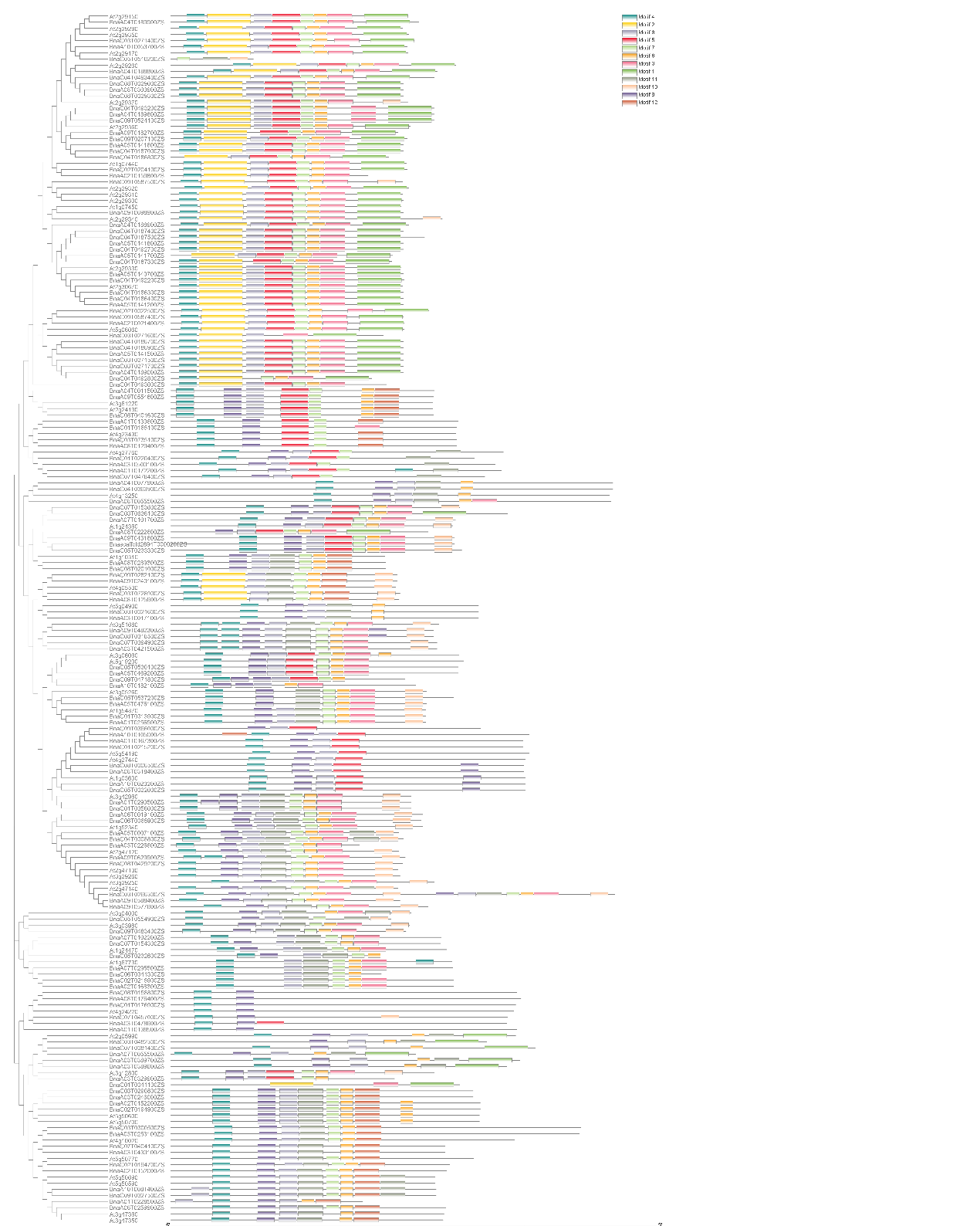


**Figure S1. Conservative motifs of the *BnaSDR* gene family**


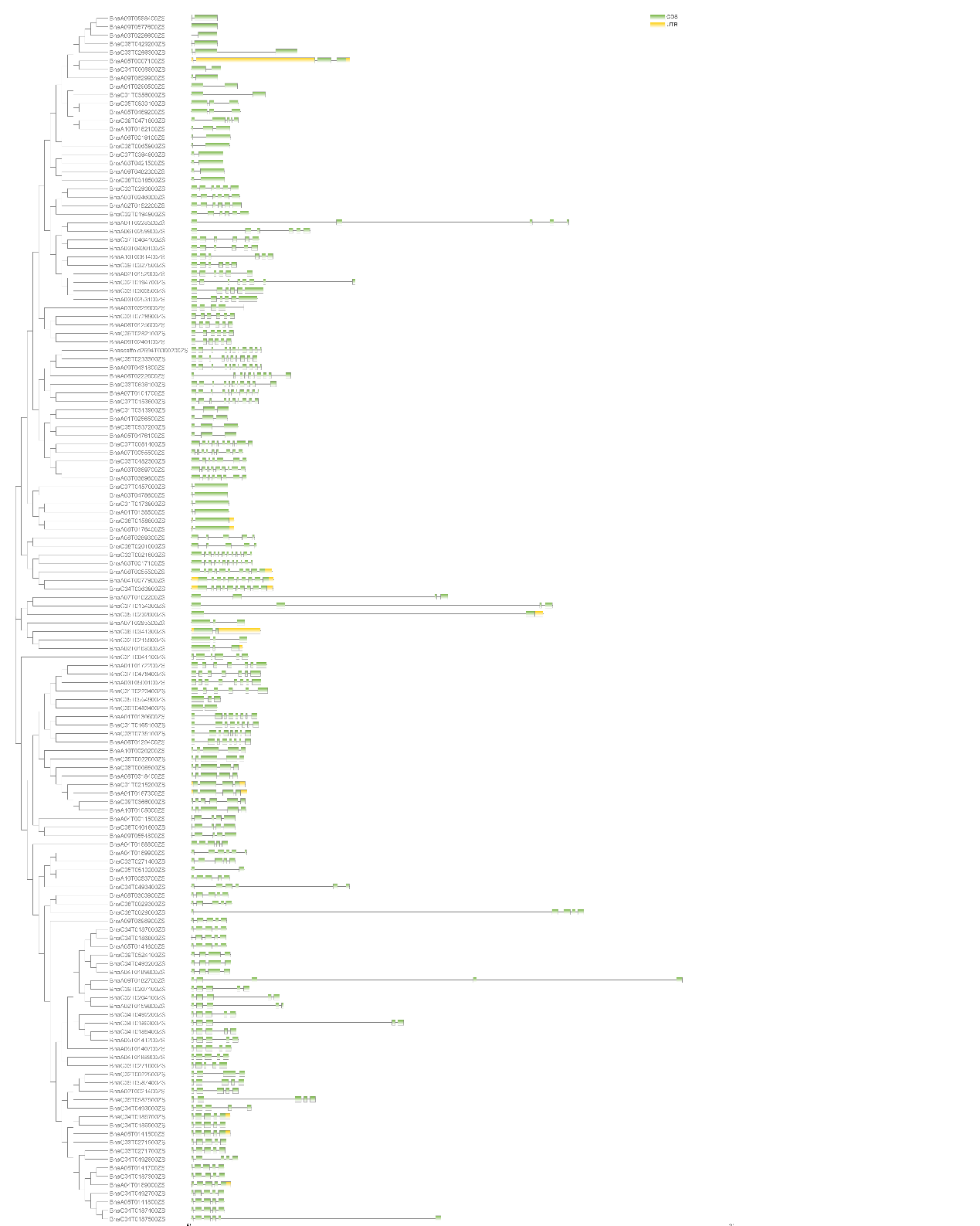


**Figure S2. gene structure of *BnaSDR* gene family**


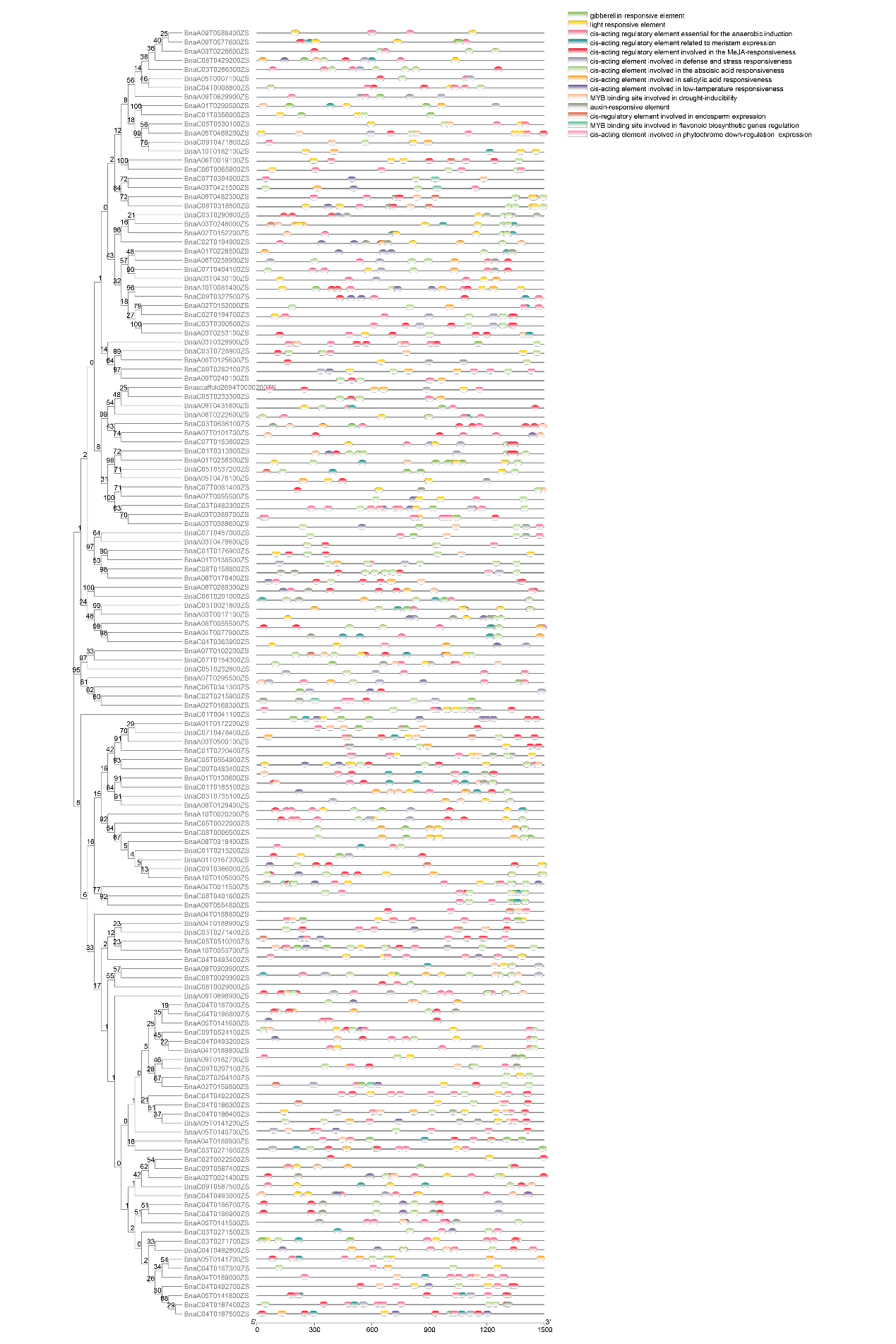
**Figure S3. cis-acting element of *BnaSDR* gene family**

**
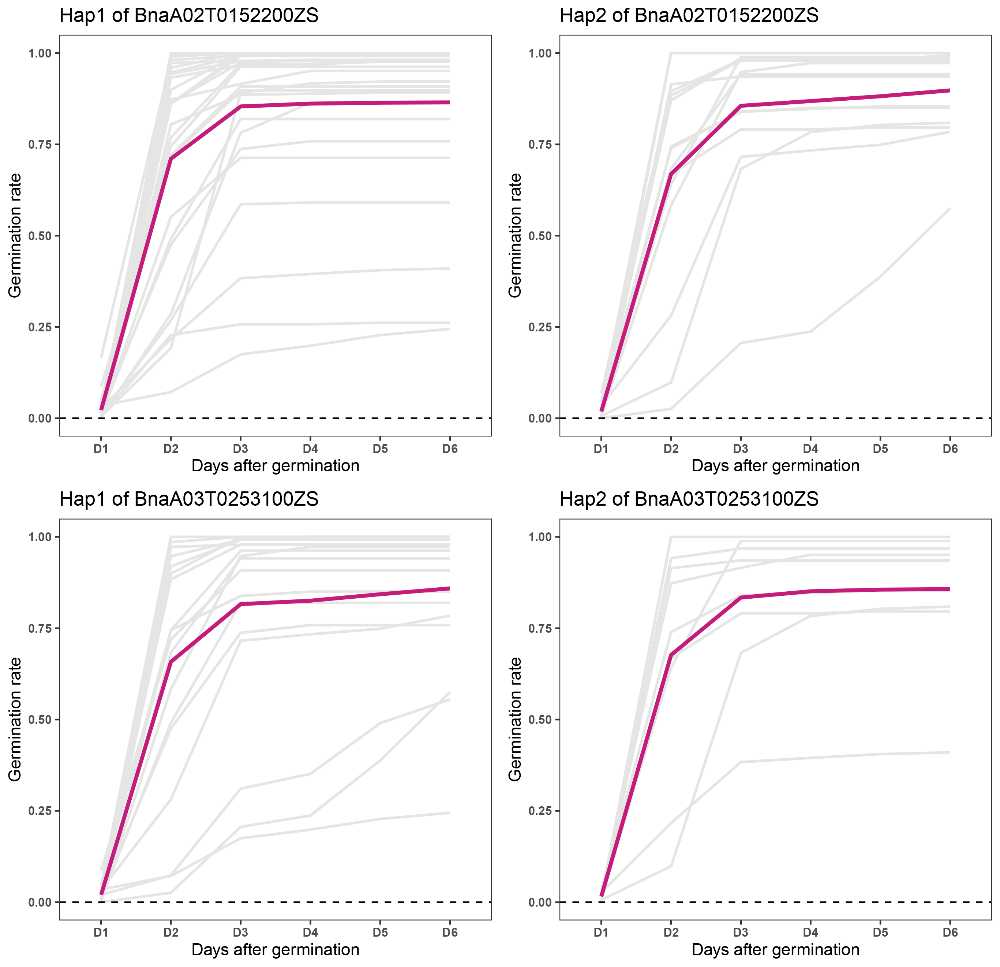

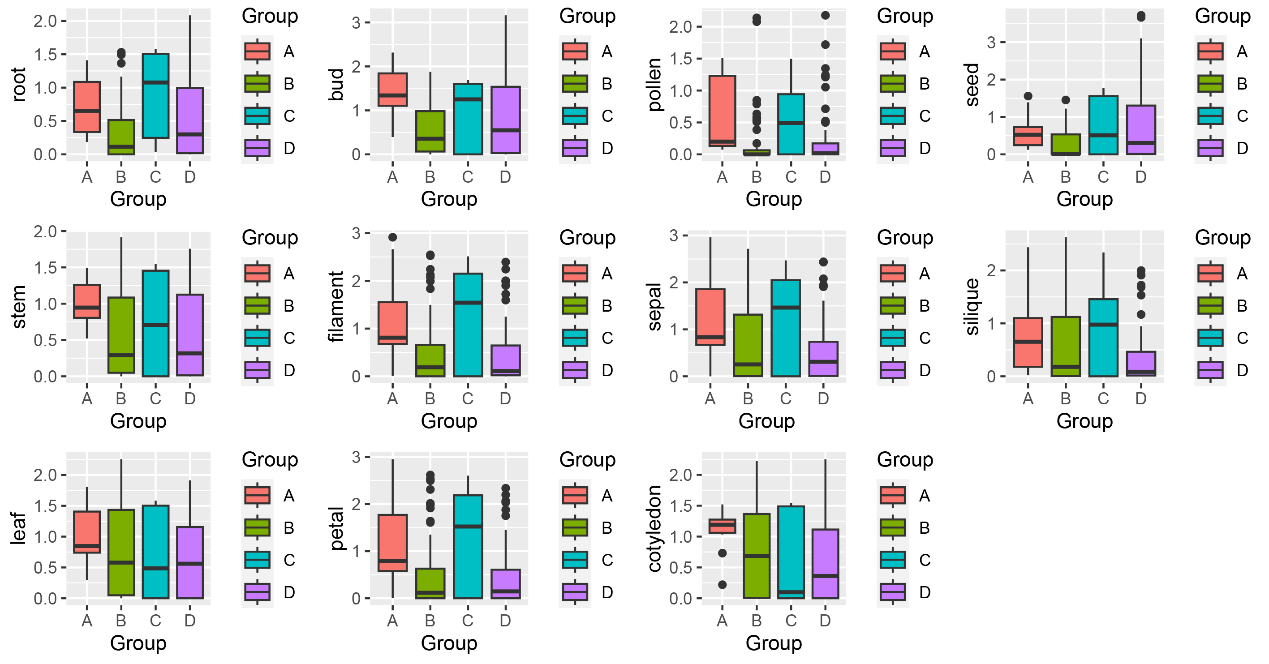
Figure S4. *BnaSDR* genes expression of *B. napus***

**Figure S5. Effects of *BnaA02T0152200ZS* and *BnaA03T0253100ZS* variation on seed dormancy in *B. napus***
